# Learning Task-Related Activities From Independent Local-Field-Potential Components Across Motor Cortex Layers

**DOI:** 10.3389/fnins.2018.00429

**Published:** 2018-06-26

**Authors:** Gonzalo Martín-Vázquez, Toshitake Asabuki, Yoshikazu Isomura, Tomoki Fukai

**Affiliations:** ^1^Department of Systems Neuroscience, Cajal Institute-CSIC, Madrid, Spain; ^2^Lab for Neural Coding and Brain Computing, RIKEN Center for Brain Science, Wako, Japan; ^3^Department of Complexity Science and Engineering, The University of Tokyo, Kashiwa, Japan; ^4^Brain Science Institute, Tamagawa University, Tokyo, Japan

**Keywords:** reservoir computing, recurrent network model, force learning, independent component analysis, gamma oscillations, theta oscillation

## Abstract

Motor cortical microcircuits receive inputs from dispersed cortical and subcortical regions in behaving animals. However, how these inputs contribute to learning and execution of voluntary sequential motor behaviors remains elusive. Here, we analyzed the independent components extracted from the local field potential (LFP) activity recorded at multiple depths of rat motor cortex during reward-motivated movement to study their roles in motor learning. Because slow gamma (30–50 Hz), fast gamma (60–120 Hz), and theta (4–10 Hz) oscillations temporally coordinate task-relevant motor cortical activities, we first explored the behavioral state- and layer-dependent coordination of motor behavior in these frequency ranges. Consistent with previous findings, oscillations in the slow and fast gamma bands dominated during distinct movement states, i.e., preparation and execution states, respectively. However, we identified a novel independent component that dominantly appeared in deep cortical layers and exhibited enhanced slow gamma activity during the execution state. Then, we used the four major independent components to train a recurrent network model for the same lever movements as the rats performed. We show that the independent components differently contribute to the formation of various task-related activities, but they also play overlapping roles in motor learning.

## Introduction

While task-related neural activities have been studied in different layers of cortical microcircuits (de Kock and Sakmann, 2009; Isomura et al., 2009; Harris and Mrsic-Flogel, 2013; Masamizu et al., 2014; Manita et al., 2015; Takeda et al., 2015), direct recordings of presynaptic inputs to local cortical circuits are still technically challenging in behaving animals. The lack of input information makes it difficult to address how inputs from different brain regions contribute to the learning of task-related cortical activities. Here, we ask this question by using the local field potentials (LFPs) recorded from the motor cortex of the rats performing a voluntary sequential arm movement (Isomura et al., 2009).

The major sources of LFP activity are widely thought to be synaptic inputs to local cortical areas. Synaptic inputs from different brain regions generally project to different layers of local cortical circuits. To segregate inputs to the motor cortex, we conducted independent component analysis (ICA) on the LFP data recorded at different cortical depths. If neural activities in different regions targeting the motor cortex are partly correlated with one another, the components extracted by ICA would not represent exact inputs from different brain areas. However, they can be, at least approximately, regarded as independent inputs converging to the primary motor cortex through multiple synaptic pathways.

First, we investigated how the independent components (ICs) are related to oscillations in various frequency bands. Ample evidence suggests that oscillations at various frequencies and their cross-frequency couplings play an active role in neural circuit functions of various brain regions (Colgin et al., 2009; Canolty and Knight, 2010; Le Van Quyen et al., 2010; van der Meer et al., 2010; Fujisawa and Buzsáki, 2011; Yamamoto et al., 2014). In the motor cortices of human (Gaetz et al., 2010; Yanagisawa et al., 2012) and non-human primates (van Wijk et al., 2012), behavioral phase-dependent shifts were shown between beta-band (15–30 Hz) and gamma-band (40–80 Hz) activities. In the rat motor cortex, slow gamma oscillation (30–50 Hz) was dominant during lever hold or preparatory periods, whereas fast gamma oscillation (60–120 Hz) was enhanced during movement execution (Igarashi et al., 2013). Our analysis based on ICA enables us to study minor oscillatory components that were previously missed.

Second, we recruited reservoir computing for exploring the roles of the ICs in motor learning. A dynamical system consisting of a recurrent neural network and readout units is called reservoir computing when readout connections, but no other connections including recurrent connections, are modifiable. In this system, the recurrent network is referred to as reservoir (Schrauwen et al., 2007). We trained the network model receiving the independent inputs to replicate the experimentally observed lever movements of the rats. The rat motor cortex shows several functional subtypes of neurons, i.e., hold-related, pre-movement, movement-related, and post-movement neurons, during the push-pull-hold movement (Isomura et al., 2009), and their relative ratio in population is different between the superficial and deep cortical layers (Igarashi et al., 2013). Results of our modeling study clarify whether the contributions of different ICs to the formation of these functional subtypes are similar or different, giving interesting insight into input- and layer-specific motor information processing.

## Materials and methods

### Experimental procedure and recording

All experiments were performed in accordance with animal protocols approved by the Experimental Animal Committee of the RIKEN Institute. All data analyzed in this study were reutilized from the 12 rats analyzed previously (Isomura et al., 2013). Briefly, head-fixed adult Long-Evans rats (*n* = 12; male 150–250 g; SLC) learnt to hold a lever for at least 1 s and then pull the lever to obtain a drop (0.01 ml) of 0.1% saccharin water for >60% of a full lever shift. After the training, a one-shank 16-channel silicon probe, which had two sets of tetrode-like electrodes (at the tip and 800 μm above it) for multineuronal activity and eight electrodes for LFP separated linearly by 150 μm (LFP8 + TetrodeSD; NeuroNexus Technologies), was inserted up to 1,200 or 1,600 μm into the forelimb area of the motor cortex (Sampling rate, 20 kHz; final gain, 2000; original band-pass filter, 0.5 Hz to 10 kHz). Spike events were isolated from the multineuronal activity with the semiautomatic spike-sorting software, EToS (Takekawa et al., 2012), and the spike clusters were manually manipulated to refine single-neuron clusters with the clustering software Klusters and NeuroScope (Hazan et al., 2006). Regular-spiking (RS) or fast-spiking (FS) neurons were classified according to the spike width (Barthó et al., 2004; Sirota et al., 2008; Saiki et al., 2014).

### Analysis of LFPs and spike phase-locking

The LFP consists of a mixed signal contributed mainly by the electric fields produced by the transmembrane currents elicited by the different synaptic inputs onto postsynaptic neurons (Elul, 1971; Buzsáki et al., 1983, 2012; Nunez and Srinivasan, 2006). To separate the different sources that contribute to the LFP we employed an ICA, a subclass of blind source separation techniques (Comon, 1994; Bell and Sejnowski, 1995; Hyvärinen et al., 2001, 2004). ICA is able to find statistically independent sources from a linear mixture, or as independent as possible (Hyvärinen and Oja, 2000). When applied to LFPs recorded by an array of electrodes distributed in the brain it can separate stable patterns of activity that are segregated in space (Hutchinson et al., 2010; Fernández-Ruiz and Herreras, 2013; Herreras et al., 2015). Applying ICA to linear profiles of LFPs spanning certain structure of the brain results in the extraction of different sources of activity with characteristic spatial distribution that can be attributed to known anatomical pathways; as it has been demonstrated in the hippocampus (Korovaichuk et al., 2010; Makarov et al., 2010; Fernández-Ruiz et al., 2012a,b, 2013; Martín-Vázquez et al., 2013, 2016; Benito et al., 2014, 2016; Schomburg et al., 2014). The use of ICA in LFP analysis has also been useful in other structures as the Lateral Septum (Martín-Vázquez et al., 2016), Lateral Geniculate Nucleus (Makarova et al., 2014) and Cerebral Cortex (Whitmore and Lin, 2016).

As the LFP is produced mainly by synaptic currents we assume that the sources are stable in space as it correspond to the transmembrane currents fixed in different dendritic domains determined by anatomy (Nunez and Srinivasan, 2006; Buzsáki et al., 2012). Thus we are assuming spatial independence for the signal's sources without any temporal constrains (i.e., spatial ICA; Stone, 2004), that allow us to perform temporal and coherence analysis between the extracted components (Bell and Sejnowski, 1995; Hyvärinen et al., 2004; Choi et al., 2005; Schomburg et al., 2014). In the case of spatially identical sources or spatially different sources that are perfectly coherent no separation of sources can be obtained with ICA (Makarova et al., 2011; Martín-Vázquez et al., 2016). But even in situations of highly correlated activity, as in the case of coupled oscillations in feedback/feedforward circuits in CA1 (Schomburg et al., 2014) and CA3 (Martín-Vázquez et al., 2016), ICA can separate different sources successfully.

The ICA model assumes that the original sources are mutually independent components that are stationary in space, mix linearly and instantaneously and have non-Gaussian distributions (Brown et al., 2001; Hyvärinen et al., 2004). Spatial stationarity of the sources of LFP is assured by the fixed synaptic input domains, as explained above. The mixture of the sources must be linear and instantaneous, as it can be assumed for the superposition of the electric fields elicited by the ionic transmembrane currents in the extracellular space in a quasistationary approximation (Plonsey and Heppner, 1967; Nunez and Srinivasan, 2006). Finally, the sources should have activation strengths with non-Gaussian distribution, which is the case for brain dynamics (Hyvärinen et al., 2010; Hyvärinen, 2012; Teramae et al., 2012; Buzsáki and Mizuseki, 2014; Omura et al., 2015).

Different ICA algorithms that are theoretically equivalent (Hyvärinen and Oja, 2000; Choi et al., 2005) and give similar results in LFP analysis (Makarov et al., 2010) have been developed. In the present study we employed “runica,” as implemented in the EEGLAB toolbox (Delorme and Makeig, 2004), a widely used method based on the logistic infomax ICA algorithm (Bell and Sejnowski, 1995) with the natural gradient (Amari, 1998). This algorithm have been extensively used in LFP analysis (Korovaichuk et al., 2010; Makarov et al., 2010; Makarova et al., 2011; Fernández-Ruiz et al., 2012a). When ICA is applied to LFPs recorded by contiguous electrodes, let be $LFP\left(t\right)={\left\{LFP_{k}\left(t\right)\right\}}_{k=1}^{8}$ (where the rows represents the *k*-electrodes and the columns each time instant *t* of the recording) we obtained *N* independent components (ICs), where N is the number of electrodes. Each IC is described by its spatial weight or distribution, *V*_*n*_ (referred also as voltage loading as explained below), that reflects the contribution of the IC to the LFP at each electrode; and its time course, *s*_*n*_(*t*), that reflects the temporal dynamics for each IC. The weighted sum of the *N*-components gives the original LFP, i.e., $LFP\left(t\right)=\overset{N}{\underset{n=1}{\sum }}V_{n}s_{n}\left(t\right)$ (Makarov et al., 2010). Specifically for “runica,” applying ICA to the LFPs results in an unmixing matrix *W* such that *WLFP* (*t*) = *s*(*t*), where the rows represent the time course of each IC, and the inverse of *W* (the so called mixing matrix) describes the spatial weights, such that *W*^−1^ = *V*, where the columns represent the spatial weights of each IC (pseudoinverse of *W* need to be used when the dimensionality is reduced previously; Delorme and Makeig, 2004).

Before applying ICA, the signal was preprocessed in various steps. We performed a principal component analysis (PCA) to reduce the dimensionality of the signal keeping 99.0% of the original LFP variance. This preprocessing diminishes the presence of noisy weak components and stabilizes and accelerates the convergence of ICA algorithm (Makarov et al., 2010; Makarova et al., 2011). Centering and whitening the signal is also common preprocessing step that reduce the computational complexity of the analysis without loss of statistical consistency (Hyvärinen and Oja, 2000; Chen and Bickel, 2005).

The knowledge of the temporal dynamics and spatial distribution of the ICs allow us to characterized them by its relative contribution to the LFP, which takes into account the temporal variance and the extension of the spatial weight along the recording sites (Makarova et al., 2011)

(1) $$\begin{array}{r} W_{n}=\frac{||V_{n}|{|}^{2}\;var\;\left(s_{n}\right)}{\sum_{m\;}||V_{m}|{|}^{2}\;var\left(s_{m}\right)}. \end{array}$$

To study the temporal and frequency relationship between ICs we reconstructed for each IC a virtual LFP by multiplying the spatial distribution (*V*_*n*_) by the time course [*s*_*n*_*(t)*], allowing us to study the ICs independently (i.e., as if each IC were active alone). This virtual LFP also allowed us to elude the ambiguity problem of spatial weights and time courses obtained with ICA, which are given in arbitrary units (Hyvärinen and Oja, 2000; Korovaichuk et al., 2010; Makarov et al., 2010). For the subsequent analyses we used the reconstructed LFP of the electrode with maximum amplitude in absolute value for each IC (*n* = 1, 2, 3, 4):

(2) $$\begin{array}{r} IC_{n}\left(t\right)={V_{n}^{\max}s}_{n}\left(t\right), \end{array}$$

where $V_{n}^{\max}$ correspond to the electrode *k* for which $\underset{k\;\epsilon \;\left[1,8\right]}{\max}$$\left|V_{n}^{k}\right|$.

To compare and classify the components between rats we use the spatial weights, that ultimately corresponds to transmembrane current distributions and hence it must be stable. We used the distance measure of the spatial distribution defined Makarov et al. (2010)

(3) $$\begin{array}{r} d\left(V_{n},V_{m}\right)=1-\frac{\left|\langle V_{n},V_{m}\rangle \right|}{||V_{n}||||V_{m}||}, \end{array}$$

(4) $$\begin{array}{r} \langle V_{n},V_{m}\rangle =\int_{\Omega }V_{n}V_{m}+\kappa {\nabla V}_{n}{\nabla V}_{m}+\kappa^{2}{\nabla^{2}V}_{n}{\nabla^{2}V}_{m}dx, \end{array}$$

(5) $$\begin{array}{r} ||V_{n}||=\sqrt{\langle V_{n},V_{n}\rangle }, \end{array}$$

between a pair (*n*,*m*) of ICs, where *d* ∈ [0, 1] due to the Cauchy-Schwarz inequality. In our case we set the dimensional constant in κ = 150 μm^2^, to magnify the spatial derivatives differences due to large inter-electrode distance. To find different groups between the set of voltage loadings, we used hierarchical clustering using the Ward's method (minimum variance algorithm) with an Euclidean metric for the distance.

The specific spatial weights of each component (V_n_) are equal to the instant depth profiles of the proportional voltage among sites of recording, i.e., the spatial loading of voltage (Makarov et al., 2010). As the multi electrode was inserted into the motor cortex spanning all layers from surface, the knowledge of the position of each recording site allow us to discriminate independent activity with cell layer accuracy (Schomburg et al., 2014). However it is important to stress that the ability of ICA to decompose the LFP into independent sources of activity depends on each particular region of the brain and the characteristics of the LFP produced there (Fernández-Ruiz et al., 2013; Benito et al., 2014; Martín-Vázquez et al., 2016). Only those synaptic inputs with enough synchrony, postsynaptic transmembrane current magnitude and spatial clustering produce measurable LFPs, optimal for ICA decomposition. Highly stratified axon terminals and parallel anatomical arrangement of the principal cells in the CA1 region of hippocampus have been demonstrated to be well-suited for ICA (Benito et al., 2014; Schomburg et al., 2014). But it also proved useful in other structures as the CA3 region (Martín-Vázquez et al., 2013; Benito et al., 2016) and Dentate Gyrus of the hippocampus (Fernández-Ruiz et al., 2013; Benito et al., 2014) or even in glomerular structures (Makarova et al., 2014). The parallel anatomical arrangement of the principal cells in the motor cortex (although overlapped) and the spatial clustered relative input strength of the different synaptic input domains (Hooks et al., 2013), supported by the existence of layer dependent LFP oscillations (Igarashi et al., 2013), makes the motor cortex an appropriate structure for the use of ICA.

### Spectrum analysis

To analyze the spectral characteristics of the LFPs with high resolution we used the complex wavelet transform (CWT) using complex Morlet wavelets (Torrence and Compo, 1998). In the case of longer periods of time the power spectra of LFP were calculated with Welch's power spectral density method using a 4096-point Fast Fourier Transform (FFT).

We performed Current Source Density (CSD) analysis to localize the current sources and sinks, and approached the second derivative of the LFP with one-dimensional finite differences along the depth of the cortex (Nicholson and Freeman, 1975; Mitzdorf, 1985):

(6) $$\begin{array}{r} CSD=-\sigma \nabla^{2}u_{k}\left(t\right)≃-\sigma \frac{u_{k-1}\left(t\right)-{2u}_{k}\left(t\right)+u_{k+1}\left(t\right)}{h^{2}}, \end{array}$$

where *u*_*k*_ is the voltage in the *k*-th electrode, σ is the extracellular space conductivity (we set σ = 1) and *h* the inter-electrode distance. Since the time course of each IC is the same for all channels, we calculated the CSD-loading using the voltage loading $I_{n}=-\sigma \nabla^{2}V_{n}$ (Makarova et al., 2011). This analysis allowed us to identify volume conducted components that had a linear spatial distribution among the recording sites and a zero CSD-loading, as it can be expected from the second derivative of a straight line (Nunez and Srinivasan, 2006). An almost straight voltage profile induced by a remote source has also been observed experimentally in the hippocampus (Korovaichuk et al., 2010) and lateral septum (Martín-Vázquez et al., 2016) of rodents and glomerular structures in monkeys (Makarova et al., 2014) as well as computationally in a model of the dentate gyrus (Fernández-Ruiz et al., 2013).

For analyzing movement of the lever we defined two phases: for the hold period we used the interval from 1,000 to 500 ms before the pull onset and for the pull period the interval from 200 ms before pull onset to 300 ms after it (Igarashi et al., 2013). Because the dynamics of the LFP and spikes in the forelimb area of the motor cortex remains the same upon lever pulling with reward and without reward (Isomura et al., 2013), we pooled the reward and no reward successful lever pulls to increase the number of trials.

To analyze the phase-amplitude cross-frequency coupling between theta and gamma oscillations we used the modulation index (MI) previously described by Tort et al. (2008). We used the Hilbert Transform to obtain the theta phase (4–10 Hz) of the raw LFP and the gamma amplitude envelope of each IC for slow gamma (20–50 Hz) and fast gamma (60–120 Hz) oscillations. For extracting the theta phase we used the raw LFP in a similar manner as Schomburg et al. (2014) and Fernández-Ruiz et al. (2017), as it showed useful for analyzing ICs obtained from the LFP, in hippocampus and entorhinal cortex. LFP signals were filtered using a linear finite impulse response filter (*eegfilt* function implemented in EEGLAB; Delorme and Makeig, 2004). We binned the theta phase in 18 intervals and calculated the mean of the gamma amplitude envelope over each phase bin. To normalize the amplitude, each bin value was divided by the sum over the bins, obtaining an amplitude distribution-like function (Tort et al., 2010). We evaluated this theta-gamma coupling computing the phase-amplitude MI that measures the divergence of the obtained amplitude distribution from a zero coupling uniform distribution (Tort et al., 2010). To evaluate the statistical significance of the MI values we performed a surrogate analysis (*n* = 200 surrogates) shuffling randomly the phase and amplitude series from different trials chosen randomly (Hurtado et al., 2004; Tort et al., 2010). Assuming that the surrogates values show a normal distribution, we established a significance threshold considering *p* < 0.01 as significant.

To detect the neurons that were significantly phase-locked to the LFP we performed a Rayleigh test (*p* < 0.05) for circular distribution (Sirota et al., 2008; Berens, 2009) and evaluated the magnitude of the coupling with the ICs using the Phase Locking Value (PLV):

(7) $$\begin{array}{r} PLV=\frac{1}{N}\left|\overset{N}{\underset{k\;=\;1}{\sum }}e^{i\theta_{k}}\right|, \end{array}$$

where θ is the phase of the ICs and *N* the number of spikes.

All the analyses were performed in MATLAB (MathWorks) and all data are expressed as mean ± SEM. The *t*-test has been widely used for evaluating statistical significance in standard analysis (Benito et al., 2014; Schomburg et al., 2014) and was also employed here.

### Neural network model

Our reservoir network consists of *N*_G_ neurons and the activity of each neuron *x*_*i*_ obeys the following equation:

(8) $$\begin{array}{r} \tau \frac{dx_{i}}{dt}=\;-x_{i}+g_{G\;}\overset{N_{G}}{\underset{j\;=\;1}{\sum }}J_{ij}^{GG}r_{j}+J_{i}^{G_{z}}z+\overset{N_{I}}{\underset{\mu \;=\;1}{\sum }}J_{i\mu }^{GI}I_{\mu }, \end{array}$$

(9) $$\begin{array}{r} r_{i}={\left[\tanh\left(x_{i}\right)\right]}_{+}, \end{array}$$

where []_+_ is a threshold linear function. The activity of readout unit *z*(*t*) was calculated as the weighted sum of the activities of reservoir neurons: *z*(*t*) = **w**^*T*^**r**(*t*), where **w** is a modifiable readout weight vector. In the present simulation, *N*_G_ = 300 and τ = 50 [ms]. Each neuron in the reservoir received an input to which one of the IC1, IC2, IC3, and IC4 was randomly assigned. In some simulations, different ICs projected to approximately the same numbers of neurons without overlaps. In other simulations, all neurons received the same IC and the contributions of each IC to motor learning were separately evaluated. The inputs were normalized between −1 and +1 and were low-pass-filtered at 10 Hz because the evolution of the present rate model is not sensitive to high-frequency components of inputs. Neuron pairs were randomly connected with the connection probability *p* = 0.1, and the weights of non-modifiable recurrent connections *J*^GG^ were determined by a normal distribution with mean 0 and variance $\frac{1\;}{\left(PN_{G}\right)}$. The overall factor of recurrent inputs was set as *g*_*G*_ = 1.5 such that the pre-training network showed chaotic activity (Sussillo and Abbott, 2009). All neurons in the reservoir projected to readout unit *z*(*t*) and were projected back to by the readout unit. The feedback connections had fixed weights $J^{G_{z}}\;$, which were taken randomly from a uniform distribution between −1 and +1. Synaptic weights to the readout were modifiable and trained by FORCE learning algorithm as in Sussillo and Abbott (2009).

### Simulations and data analysis

Training data for arm trajectory and LFP data were obtained in Isomura et al. (2009). In the behavioral task, rats spent the majority of task period for lever-hold and generated movements only during a small portion of the task period. To enable efficient learning of movements, we only used data segments containing lever pull in each trial, namely, from 1 s before to 500 ms after lever pull onset. The data set used for learning was obtained from 18 trials. After learning, activity of each neuron in the reservoir was averaged over the 18 trials and normalized between its minimum and maximum values. Then, activity was categorized into five distinct functional subtypes as in Isomura et al. (2009). Briefly, Movement-related activity was a phasic activation during movements, whereas Hold-related and Movement-off neurons exhibited a phasic decrease of activity during movements. The latter two types of functional activity were not distinguished in this study. Pre-movement was a phasic activation starting earlier than 500 ms before movement onset and rapidly dropping by more than the half of its peak activation after movement onset. Post-movement was a phasic activation starting from movement onset and dropping within 350 ms from the onset. Activity profiles that were not categorized into any of these functional subtypes were categorized as “others.” Phasic activity was defined as activity of each neuron that increased or decreased beyond μ ± 3σ for more than 60 ms, where μ and σ stand for the average and standard deviation of its activity during 1,000–250 ms before movement onset, respectively. In Figure 5C, neurons were sorted according to the serial order of activation time, which was calculated as

(10) $${\hat{t}}_{i}=\frac{T}{\pi }\arg\left[\frac{\sum_{t^{\prime }}^{T}{\bar{r}}_{i}\left(2t^{\prime }-1000\right)\exp\left(i\frac{2\pi^{\prime }}{T}\right)}{\sum_{t^{\prime }=1}^{T}{\bar{r}}_{i}\left(2t^{\prime }-1000\right)}\right]\;\left[\text{ms}\right],$$

where *r*(*t*) is the normalized average response of each cell, *T* = 750 ms and the activation time was adjusted such that it falls within the range [−1,000, +500] ms.

## Results

### Independent components of LFPs in the motor cortex

We recorded LFP signals at eight different depths of the motor cortex (layer 2/3 to layer 6) in rats performing an alternate-reward forelimb movement task. To identify independent sources of activity contributing to the LFP, we applied ICA to the raw LFP from the whole recording and obtained spatial voltage distribution (referred to as spatial loading) and time course of each independent component (IC). The results obtained were qualitatively the same when applying ICA only to the movement periods, so we show the results for the whole recording analysis because it can be extracted more information of the independent activity. In 10 of the 12 analyzed animals we found four major ICs (i.e., IC1, IC2, IC3 and IC4), with different and recognizable time dynamic and layer stratification along the motor cortex. In the other two rats we could not obtain a clear IC3 and IC4, so we excluded these rats from the rest of the analysis. The ICs obtained ultimately represent independent activity of the LFP that show different layer dependences along the depth of the motor cortex. Overall, our results are consistent with those previously reported in Igarashi et al. (2013) for the same set of rats without using ICA. As shown below, however, ICA decomposition of LFPs enabled us to accurately clarify the layer-dependence of the independent activity in the LFP of the motor cortex.

**Figure 1:**
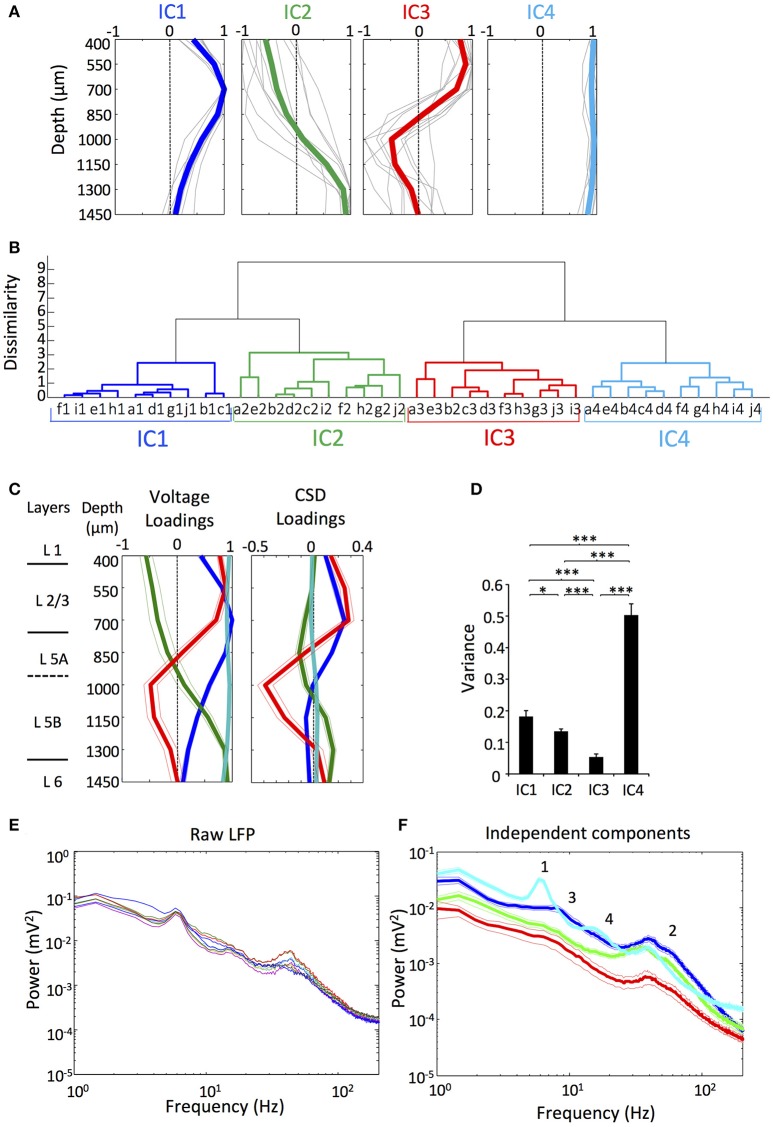
There were four main ICs of the LFP in the motor cortex. **(A)** Normalized voltage loadings of the four main ICs. Colored lines represent the mean value and gray lines represent the voltage loadings of different rats (*n* = 10). **(B)** Cluster analysis of the voltage loadings from 10 rats (a-j) established four different groups corresponding to the four different ICs. *n* = 10. **(C)** Mean Normalized voltage loadings (Left) and CSD loadings (Right) of the ICs from all the animals and the previously obtained scheme of the approximate layer-depth correspondence for the primary motor cortex (Isomura et al., 2009; Hooks et al., 2013). *n* = 10. **(D)** The variance of the ICs showed the differential contribution to the LFP. *n* = 9. (^*^/^***^p < 0.05/0.01/0.001; *t-*test). **(E)** Representative power spectra of the raw LFP in the motor cortex of an animal as was established previously by Igarashi et al. (2013). Each colored line represents an electrode with different depth. (F) Mean power spectra of the ICs. Numbers 1–4 indicate various relevant features of the profiles (see section Results). *n* = 9.

The four ICs exhibited certain relationships across rats recognizable by visual inspection of the voltage loadings, suggesting that the components were stable (Figure 1A). To confirm the identity of the ICs and the correctness of their classification more rigorously, we measured the distance between the voltage loadings of the ICs between all the rats, evaluating the pair-wise dissimilarity. We then constructed a dendrogram based on the dissimilarity information. The dendrogram in Figure 1B shows the hierarchical binary cluster tree of the distances, which demonstrates that the four ICs were successfully grouped between different rats in four different branches with some dispersion due to small variability in experimental conditions (i.e., different depth of the electrode, artifacts, and noises).

The spatial loading of each IC ultimately represents the strength of its activity in the different layers of the motor cortex, so an exhaustive study is required in order to describe the layer-dependence. As we cannot assign the possible inhibitory/excitatory nature of the LFP activity ascribed to each IC without performing local injection of neurotransmitter receptors blockers (Martín-Vázquez et al., 2013) we focused only in the shape of the curve of the spatial loading without considering the polarity (Makarov et al., 2010). As it can be expected, ICs had a differential contribution to the LFP along the depth axis of the cortex (Figure 1C). IC1 presented a maximum in the voltage loading at ~700 μm around layer 2/3 and did not reverse along the depth axis. IC2 showed a maximum in the voltage loading at ~1450 μm corresponding to layers 5B and 6, and reversed the potential around ~1,000 μm. In the motor cortex, layer 5 is classified into 5A and 5B (Ueta et al., 2014). Previously, we measured the physiological depth of juxtacellularly recorded neuron, and determined the layer (5A or 5B) where it was visualized histologically. We found the border between the two layers at about 1,100 to 1,200 μm from the cortical surface (Isomura et al., 2009). IC3 showed a maximum in the voltage loading above IC1, still within layer 2/3, and reversed at the same depth as IC2 did. IC4 showed a linear spatial loading, suggesting that it possibly reflects a volume conducted component that came from other regions of the brain.

We also obtained the spatial loading of current source density (CSD) for each IC to localize the currents that underlie the spatial voltage loading (Figure 1C). IC4 presented a linear CSD loading around zero that supports a volume conduction origin. Small deviations along the depth axis possibly arose from contamination with artifacts or noise, though it can alternatively represent a local source of small magnitude. The other three components showed clear reversals spanning different depth of the cortex. Relative contributions of the different ICs to the variance of LFP during the whole recording session were significantly different (Figure 1D). In fact, IC1, IC2, and IC3 represent signals that were arranged in a descendant order of the variance. Somewhat unexpectedly, IC4 was the dominant component of the LFP, implying that the volume conducted component accounted for most of the variance of the LFP recorded in the motor cortex, as it has been reported previously using the same methodology, i.e., applying ICA to LFP of the cortex (Whitmore and Lin, 2016).

**Figure 2:**
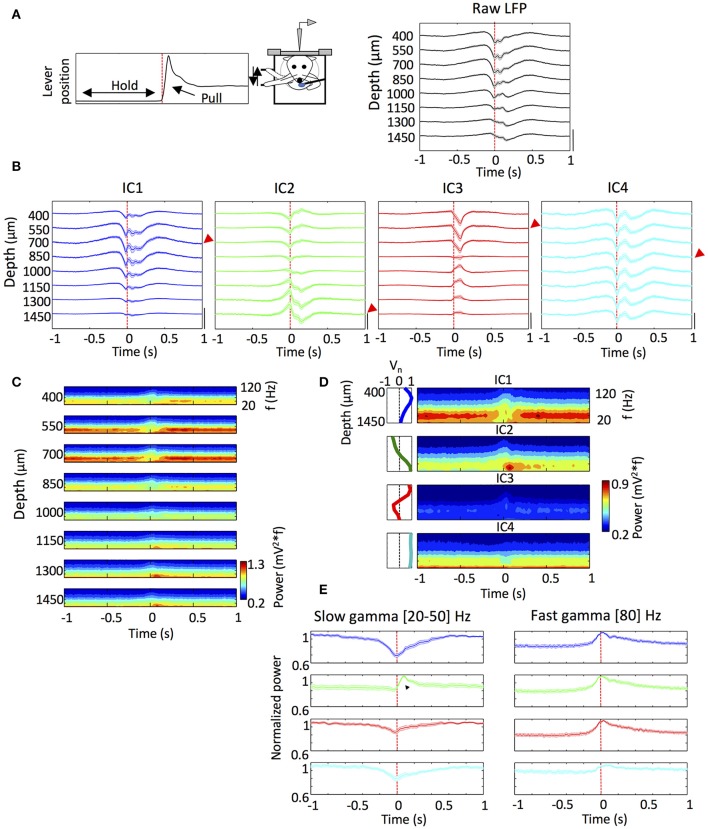
The ICs exhibited different dynamics during the performing of a reward motivated motor task. **(A)** Scheme of the head restrained rats performing the reward motivated forelimb movement task and a representative average of the lever position from an animal indicating hold and pull periods (Top) and mean of the lever pull onset-triggered average of the raw LFP (averaged from 1,200 to 2,000 trials in each rat) (Bottom). *n* = 10. **(B)** Same as **(A)** but for the reconstructed LFP for each IC. The red arrows indicate the electrode with the maximum amplitude in absolute values for each IC that was used below (See section Materials and Methods). *n* = 10. Amplitude calibration in **(A,B)**: raw LFP, 1 mV; ICs, 0.5 mV. *n* = 10 rats. **(C)** Pull onset-triggered average of the Wavelet power spectrum of the raw LFP at different depths from a representative animal. **(D)** Same as **(C)** but for the ICs from the same animal. Inset: scheme of the voltage loading of each IC. **(E)** Time evolution of the slow gamma (20–50 Hz) and fast gamma (80 Hz) powers of the ICs around the lever pull onset. The traces correspond to the power of the wavelet power spectrum for a given frequency (between 20 and 50 Hz) where the IC exhibited a maximum in the power. The values of the frequencies at which each IC had a maximum differed between ICs (see section Results). The black triangle shows the sharp slow gamma power increase of the IC2 after the pull onset. Fast gamma powers were measured at 80 Hz for each IC. *n* = 10 rats. In all the panels the time 0 and the dashed red line mark the lever pull onset.

Rigorously, ICs are comprised of time courses that vary in the amplitude in the different sites (Makarov et al., 2010), so in order to select representative signals for the subsequent analysis, we used the reconstructed LFP of the electrode with the maximum amplitude for each IC (see section Materials and Methods and Figure 2B, below). In Figure 1E, we show the power spectra obtained from the representative raw LFPs from an animal (as was established previously by Igarashi et al., 2013) and those obtained from the means of the individual ICs (Figure 1F). In the LFP power spectra, the profiles were very similar between the electrodes, while the ICs power spectra showed heterogeneous profiles, presumably because of the different synaptic origins. The theta peak of the raw LFP (around 5–8 Hz; 1 in Figure 1F) was ascribed mostly to the IC4 whereas the gamma peak (around 20–100 Hz; 2 in Figure 1F) appeared in all the components, being the power of IC3 notably lower. IC1 also showed a small and broad hump in lower frequencies (around 3–20 Hz; 3 in Figure 1F) including the theta band, while IC4 had a small peak around 10–20 Hz (4 in Figure 1F).

### Temporal relationships of ICs with motor behavior

We were interested in exploring the dynamics of the different ICs during reward-motivated movement task. To this end, we averaged the LFP and the ICs over trials after aligning the signals to the pull movement onset (time 0 in Figures 2A,B). The averaged LFP signal showed a complex pattern starting before the pull movement onset and decaying hundreds of milliseconds after the onset (Figure 2A). The averaged IC signals showed similar patterns for IC1, IC2 and IC4, starting or ending about a hundred to several hundred milliseconds before or after, respectively, the pull onset (Figure 2B). In contrast to these components, the IC3 showed the peak of maximum amplitude about 100 ms after the pull onset, which approximately coincides with the time of reward delivery.

We then analyzed information about the frequencies involved in the dynamics of each IC by averaging the wavelet power spectra of the LFP as before (Figure 2C). As shown previously (Igarashi et al., 2013), the slow and fast gamma oscillations appeared during distinct movement states: the slow gamma band (20–50 Hz) was generally dominant during holding period; the fast gamma oscillation (60–120 Hz) started to appear about 100 ms before the onset of lever pull and lasted during movement execution. Similar tendency was also seen in the average wavelet power spectra of the ICs (Figure 2D).

However, the averaging of ICs revealed remarkable differences in time evolution between them. In Figure 2E, we measured the maximum powers of the averaged wavelet power spectra separately for slow gamma ([20, 50] Hz) and fast gamma (80 Hz). A sharp enhancement of fast gamma oscillation was commonly seen in IC1, IC2 and IC3 when aligned with pull movement, only differing in the maximum power (max powers 0.55 ± 0.04, 0.44 ± 0.02, and 0.28 ± 0.03 mV^2^, respectively). Slow gamma oscillation also showed noticeable changes around the pull onset. IC1 slow gamma was predominant during the lever hold (max power 1.02 ± 0.10 mV^2^) and started to decrease about 250 ms before the pull onset, and this suppression of slow gamma continued during movement execution. IC3 and IC4 showed a similar behavior, though their slow gamma powers during the lever hold were much lower (max powers 0.42 ± 0.05 and 0.79 ± 0.03 mV^2^; *p* < 0.001 and *p* = 0.021, respectively; *t*-test), and so were the decrease ratios. All these results are consistent with the previous results.

In a striking contrast, IC2 slow gamma showed a sharp increase ~100 ms after the pull onset (max power 0.85 ± 0.03 mV^2^). The increase in the slow gamma power was previously not detected (Igarashi et al., 2013), and it was concluded that all slow gamma components undergo suppression during movement. However, the present results of ICA reveal that this is not entirely true. As seen by visual inspection of the wavelet power spectra in Figure 2D, the peak frequency of IC2 slow gamma (31.9 ± 0.6 Hz) was significantly lower than the peak slow-gamma frequencies of the other ICs (36.3 ± 1.2 Hz for IC1, 36.5 ± 1.5 Hz for IC3 and 34.5 ± 1.4 Hz for IC4; *p* = 0.002, *p* = 0.004, *p* = 0.048, respectively; *t*-test).

### Couplings of ICs to slow and fast gamma oscillations

**Figure 3:**
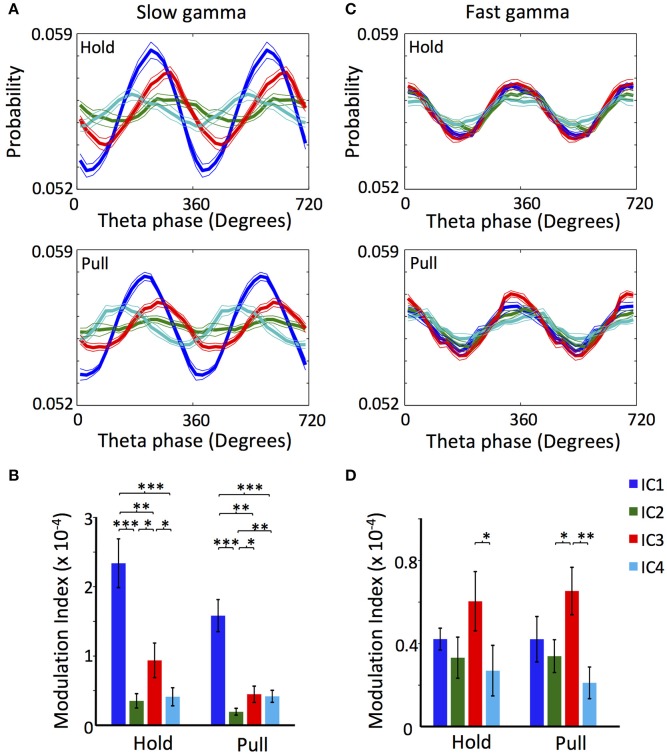
Cross-frequency coupling between theta LFP and gamma oscillations of the different ICs. **(A)** LFP theta phase-slow gamma amplitude distribution of the ICs during lever hold (Top) and pull (Bottom). **(B)** Modulation index of the LFP theta phase-slow gamma amplitude distribution of the ICs during lever hold and pull. **(C)** Same as **(A)** but for fast gamma. **(D)** Same as **(B)** but for fast gamma. The LFP used for extracting the theta phase were the one of the electrode with the highest mean power along the whole. All the analyses were performed in 1,200–2,000 trials for each rat. *n* = 10 rats. (^*^/^**^/^***^p < 0.05/0.01/0.001; *t-*test).

We further explored how the different ICs are coupled to slow and fast gamma oscillations. To this end, we checked the cross-frequency couplings between the gamma oscillations of the ICs and the LFP theta oscillation (Schomburg et al., 2014; Fernández-Ruiz et al., 2017), which is synchronized across the different layers of the motor cortex and hence provides reference time points. The theta, slow gamma and fast gamma oscillations of LFP change its absolute power during the realization of the task (Igarashi et al., 2013). For this reason and for avoiding possible spurious modulation, we used the Modulation Index analysis (MI; see section Materials and Methods) that is sensitive only to the phase of the theta signal and the relative variation in the power of the gamma oscillations along the theta phase (Tort et al., 2010). For this purpose, we constructed phase-amplitude distribution plots for slow gamma (20–50 Hz) and fast gamma (60–120 Hz) during holding and pulling, and computed the phase-amplitude modulation index. Figure 3A shows that slow gamma oscillation of IC4 preceded the oscillations of the other ICs (*p* < 0.01 Watson-Williams test measured for the theta phase with maximum probability), while IC1 preceded IC2 and IC3 (*p* < 0.01; Watson-Williams test). Furthermore, the MI of the slow gamma oscillation of IC1 was significantly higher than the rest of ICs during both holding and pulling, while IC3 showed a higher value than IC2 (Figure 3B). Interestingly, IC1 and IC3 reduced significantly the MI during pulling when compared to holding (*p* = 0.03 and *p* = 0.005, respectively; *t*-test).

There were no major phase differences in the phase-amplitude distribution of the ICs fast gamma oscillation; the four ICs showed the peak of maximum amplitude around 360° (*p* > 0.05; Watson-Williams test; Figure 3C). Values of the MIs were also similar for the fast gamma oscillations. Though IC3 exhibited significantly higher values than IC2 and IC4, the difference was not significant between IC3 and IC1 (Figure 3D). None of the ICs varied the MIs values in the holding-to-pulling transition (*p* > 0.05; *t*-test).

Spikes of 80 neurons were sorted from the superficial and deep layers of the motor cortex and the neurons were classified into regular-spiking neurons (RS; *n* = 19 and 46 for superficial and deep layers, respectively) and fast-spiking neurons (FS; *n* = 7 and 8 for superficial and deep layers, respectively) according to the width of the spikes (Isomura et al., 2009). Among the RS neurons of the superficial layers, 16, 13, 12, and 10 neurons in the slow gamma b and and 15, 8, 13, and 9 neurons in the fast gamma band were phase locked for IC1, IC2, IC3, and IC4, respectively. For both gamma bands IC1 was dominant. Among the RS neurons of the deep layers, 24, 20, 17, and 30 neurons in the slow gamma band and 21, 33, 22, and 34 neurons in the fast gamma band showed phase locking for IC1, IC2, IC3, and IC4, respectively. In this case IC4 was dominant. All the FS neurons exhibited phase locking in both gamma bands for the four ICs (*n* = 5 in superficial layers and *n* = 8 in deep layers), except that only 4 FS neurons of the superficial layers showed phase locking for IC3 in the fast gamma band.

**Figure 4:**
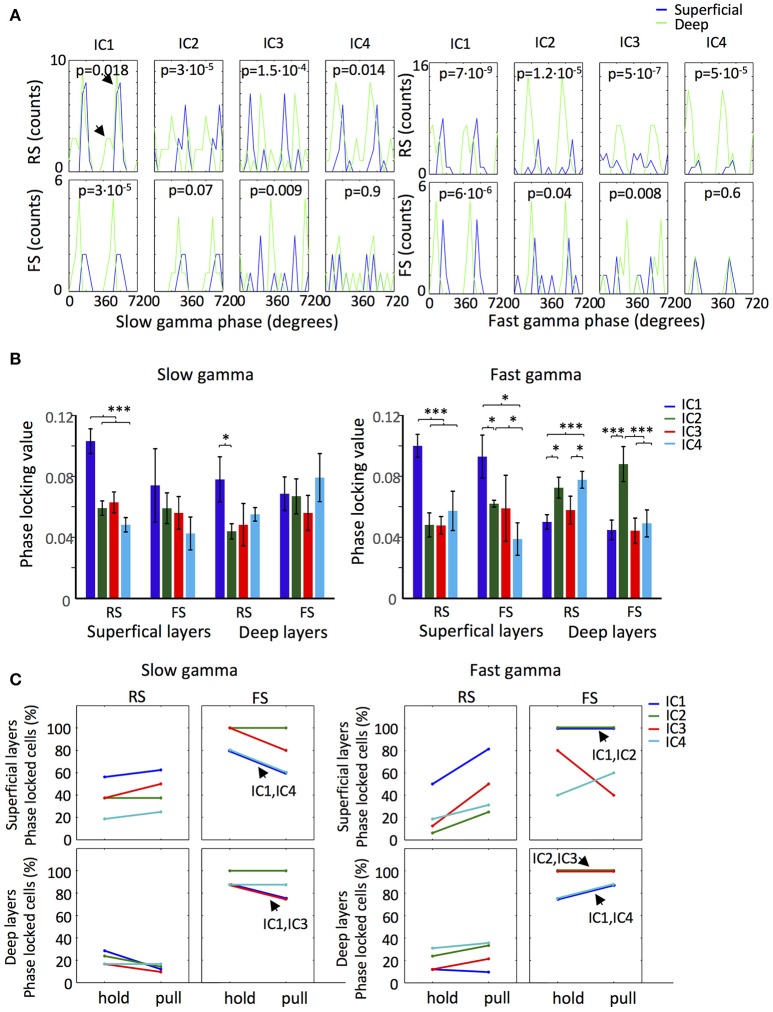
IC gamma phase locked firing of motor cortex neurons. **(A)** Distribution of the slow gamma (Top) and fast gamma (Bottom) ICs mean phases of the significantly phase locked RS and FS neurons of the superficial (Blue line) and deep (Green line) layers. *p*-values of Watson-Williams test in each panel measure the significance of the difference between the superficial and deep layers neurons for each IC for the two types of neurons. The arrows in the first panel indicate the existence of two preferred slow gamma phases. **(B)** ICs Slow gamma (Left) and fast gamma (Right) phase locking values of the RS and FS neurons from the superficial and deep layers. (^*^/^***^p < 0.05/0.01/0.001; *t-*test). **(C)** Proportion of slow gamma (Left) and fast gamma (Right) phase locked neurons during lever hold and pull. In the cases where two ICs have the same proportion and the lines overlap it is indicated by an arrow.

The phase preference of the recorded neurons displayed interesting dependences on the ICs. Neurons located in deep layers tended to fire earlier in the fast and slow gamma cycles than neurons in the superficial layers in IC1, IC2, and IC4, but conversely in IC3 (Figure 4A). Interestingly, RS neurons in the deep layers seemed to have two preferred IC1-phases in both slow and fast gamma bands, suggesting the existence of two different populations (Figure 4A; arrows). RS neurons showed particularly strong phase locking to IC1 slow and fast gamma oscillations, yielding the highest values of PLV (see section Materials and Methods) in the superficial layers (Figure 4B). FS neurons exhibited the highest PLV value in the deep layers for IC2 fast gamma oscillation.

To explore the behavioral phase dependence of phase locked firing, we analyzed the proportion of neurons phase locked to the different ICs during holding and pulling (Figure 4C). In both layers, the pattern of phase locking for each IC differed between RS and FS neurons. Notably, the number of superficial layer RS neurons phase locked to the fast gamma was increased during pulling for all the ICs. In both layers, IC2 slow and fast gamma oscillations maintained the proportion of phase locked FS neurons across the periods of holding and pulling. Thus, our results suggest that couplings between neuronal firing and gamma oscillations can vary in different ICs are different cell types.

### Roles of ICs in motor learning

**Figure 5:**
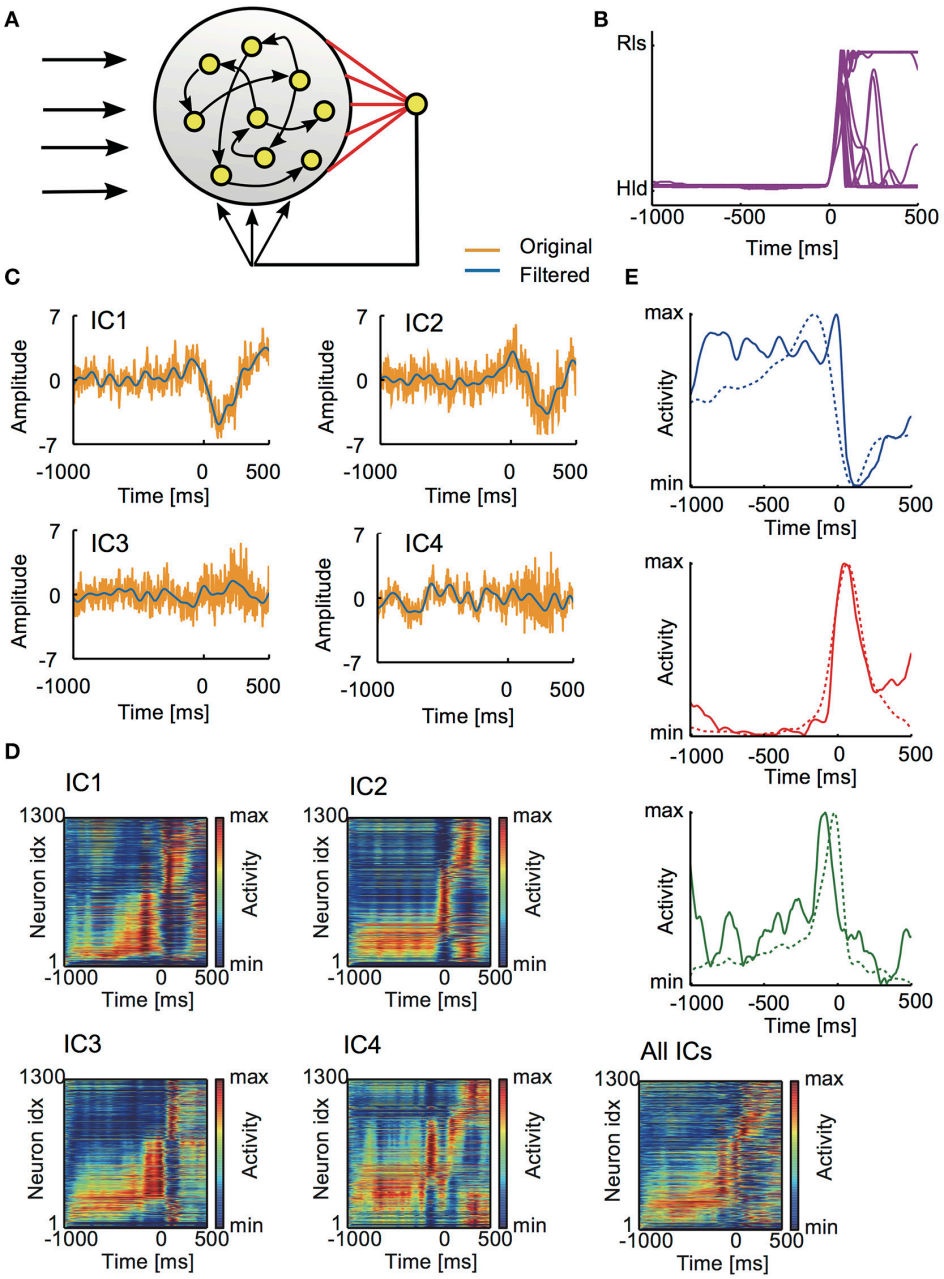
Learned activity patterns in the reservoir network model. **(A)** The model is schematically illustrated. **(B)** Lever movements in 18 trials provided teaching signals for learning. Time 0 refers to the onset of lever pull. **(C)** Typical examples of the independent components of LFP (yellow) and their low-pass-filtered versions (blue) are shown. **(D)** The average normalized responses of all neurons in five different conditions: external input to the reservoir involved all ICs or just one IC. The activities of individual neurons were averaged over repeated trials and sorted according to the onset time of activation (section Materials and Methods). **(E)** Examples of the average responses are shown for Hold-related or Movement-off (top), Movement-related (middle) and Pre-movement (bottom) neurons.

We examined whether the four ICs obtained from the rat motor cortex are sufficient for motor learning. We trained a reservoir computing model of rate-coding neurons without layer structure to generate experimentally observed arm movements (Figures 5A,B; section Materials and Methods), and evaluated the contribution of each IC to organizing functionally different neural activities. Training a realistic cortical microcircuit model is beyond the scope of this study. The reservoir received the ICs (10 Hz cut-off) as external inputs (Figure 5C) and FORCE learning was used for the training (Sussillo and Abbott, 2009). Because rate-coding neurons are insensitive to input changes faster than the membrane time constant, the ICs were low-pass filtered without changing the essential results. A related modeling study has been performed in the monkey motor cortex by using movement-preparatory activity as input (Sussillo et al., 2015). Here, we aimed at generating all functional subtypes of neurons by using the ICs as input.

The reservoir model was trained simultaneously with all four ICs or separately with each IC. The post-learning responses of individual neurons are shown for each case (Figure 5D). In all the cases, the resultant population activity contained neural responses which are similar to various task-related neurons found in experiment. Examples of the Hold-related (or Movement-off), Movement-related and Pre-movement activities learned by the network model are in Figure 5E.

**Figure 6:**
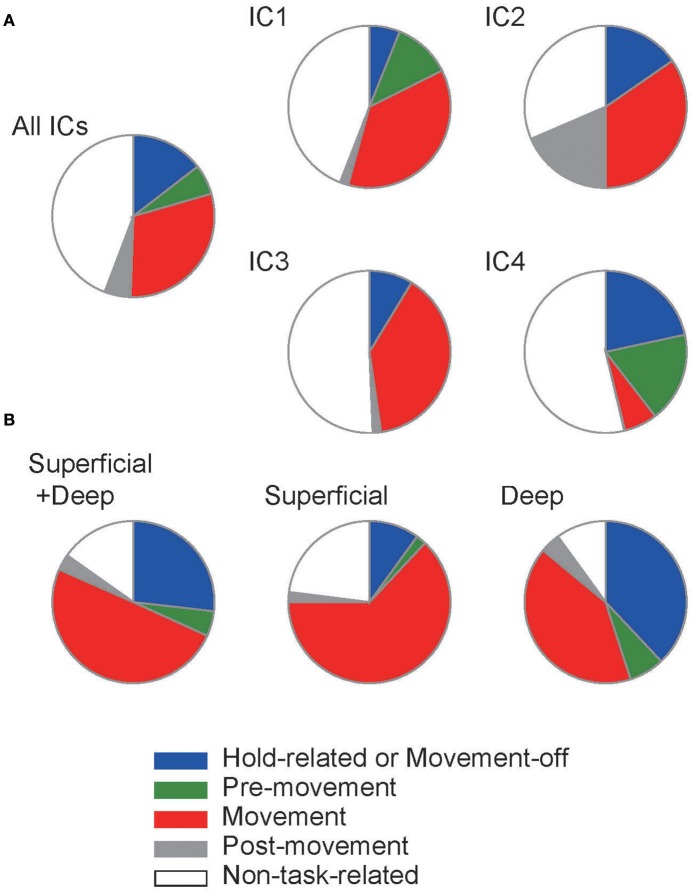
Population fraction of each functional subtype. **(A)** The population ratios of functional subtypes were calculated in the models with different settings of input. **(B)** Similar population ratios were obtained from the experimental data reported in Igarashi et al. (2013).

Depending on the input conditions, the model exhibited a different fraction of Movement-related neurons and Hold-related (or Movement-off) neurons (Figure 6A). The fractions obtained from the simulations are compared with those observed in experiment (Figure 6B, superficial plus deep layers). In the model, contributions of individual ICs exhibited interesting differences. For IC1 and IC3, the fraction of Hold-related neurons was small compared to that of Movement-related neurons, while opposite was true for IC4. In experiment, movement-related neurons were found dominantly in the superficial layer (Figure 6B), suggesting that the major drivers of the superficial layer involve IC1 and IC3. In contrast, the deep layers contained nearly identical fractions of these functional subtypes, from which a reliable assessment of the relative contributions of ICs seems to be difficult.

Overall, our results suggest that the individual ICs differently contribute to the formation of different functional subtypes of neurons. However, all of them contribute to two major subtypes, i.e., Hold-related and Movement-related neurons. Therefore, the ICs play overlapping roles in learning sequential motor behavior. While the model replicated the experimentally observed spectrum of functional subtypes, it contained a larger fraction of non-task-related neurons than experimental data, indicating the limitation of the model.

## Discussion

In the present study, we reported and characterized four main independent components in the LFP of the motor cortex (Figure 1). During the lever movement task, the ICs exhibited complex dynamics: IC1, IC2, and IC4 increased the activity before and during the movement realization, and IC3 started just at the beginning of the movement (Figure 1B). These characteristic evolution patterns of the different ICs appear most strongly in different layers of the motor cortex. The spectrum analysis of the ICs also revealed complex dynamics of their frequency patterns.

The layer dependence of the relative strength of IC1, IC2 and IC3 (see Figure 1A) exhibits a certain degree of correlations with the anatomical structure of long-range projections from distinct brain regions to the primary motor cortex. IC1 is strongest in layers 2/3 (at the cortical depth of about 700 μm) and gradually becomes weak toward deep layer 5 (layer 5B); IC2 is strongest in layer 5B and is weaker in layers 5A and 2/3; IC3 is strong in layers 2/3 and 5A, but not in layer 5B. We may compare these results with anatomical data for synaptic inputs to the rodent primary motor cortex (Hooks et al., 2013). The motor thalamus projects equally strongly to layers 2/3, 5A, and 5B of the motor cortex, while the primary somatosensory cortex and sensory thalamus project strongly to layers 2/3 and 5A, but this projection rapidly becomes weak toward the border between layers 5B and 6. These patterns of layer dependence are similar to those of the magnitude changes in IC1 and IC3. Then, the secondary motor cortex and orbital cortex project weakly to L2/3 and L5A, and strongly to L5B. This projection pattern resembles the spatial pattern expressed by IC2.

The power spectra of the LFPs show the behavioral phase- and cortical layer-dependent evolution patterns of their frequency components. In particular, we could identify a sharp increase in slow gamma activity in the deep layers of motor cortex ~100 ms after the execution of a forelimb movement. This increase was previously uncovered, solely ascribed to IC2 (Figure 2D), and not seen in other ICs (Figure 2E). This slow activity may represent a somatosensory feedback input or a top-down input from higher cortical areas (Saiki et al., 2014). In particular, it has been shown that inputs from the orbital cortex and secondary motor cortex to the primary motor cortex are related with the cognitive and volitional aspects of movements (Saiki et al., 2014). In addition, neurons in the deeper layers of the primary motor cortex project to the brainstem and thalamus (Hooks et al., 2013). Together with these observations, our results support the speculation that IC2 could be engaged in the volitional control of movements.

IC3 also allows some interpretations as it shows a peak activity after the pull movement (Figure 2B). In the behavioral task, the time of this peak activity approximately coincides with reward delivery, but IC3 is unlikely to represent a reward-related signal. As previously established (Isomura et al., 2013), the motor cortex is not involved in the coding of reward information (neither in the activity of regular-spiking and fast-spiking neurons nor in the LFP; see also Saiki et al., 2014). Consistent with this, we could not find noticeable differences in the activity of ICs between reward and no-reward conditions (data not shown). Because layers 2/3 and 5A, in which IC3 shows the strongest change, are projected to by the motor thalamus (Hooks et al., 2013) and because the thalamus is thought to monitor motor outputs (Guillery and Sherman, 2002), a likely hypothesis is that IC3 is related with some type of sensory or motor feedback of the movement.

The amplitude of each IC peaked at different phases of theta oscillation, suggesting a hierarchical coordination of the different inputs to the motor cortex (Figure 3). The phase locked firing of motor cortex neurons to slow and fast gamma oscillations exhibited large differences between ICs in phase preference, PLV, and behavioral phase dependence (Figure 4), although such differences were subtle in the fast-gamma band compared to the slow-gamma band (Figure 3). Results for IC1 suggested the presence of two different populations of deep-layer RS neurons showing different preferred phases of spiking in IC1 slow and fast gamma oscillations (Figure 4A). Such a repulsion of preferred phases may occur if these neuron groups inhibit each other via inhibitory interneurons.

The origin of IC4 seems to be volume conduction, implying that most of the contribution to the LFP of motor cortex comes from distant areas, which is in accordance with previous results using ICA in the LFP of the cortex (Whitmore and Lin, 2016). The main argument for volume conduction arises from the uniform loading of IC4 (Figures 1A,C) typical of propagated activity between two regions, due to the extension of the electric field in space (Fernández-Ruiz et al., 2013; Martín-Vázquez et al., 2016). The proximity of hippocampus (Paxinos and Watson, 2009) and the marked presence of theta oscillations (Figure 1F) show that IC4 may have contribution, at least partially, from hippocampal activity (Sirota et al., 2008), although other regions could contribute as well (Kajikawa and Schroeder, 2011). Thus, the relations found between IC4 and motor cortex theta LFP (Figure 3) and spike activity (Figure 4) should be understood as a coupling between motor cortex activity and distant activity of unknown origins.

We conducted the demixing of independent activity of each IC from a set of layer-dependent LFP signals that would express different mixtures of the ICs, relegating the sources of these ICs to speculation. Ultimately, the sources of the ICs are principally the transmembrane currents elicited by the different synaptic inputs (Nunez and Srinivasan, 2006), but to assure that each IC is pathway specific and to identify each of them with a known anatomical input require additional experiments (Herreras et al., 2015): local pharmacological injection as well as local electrical stimulation (Martín-Vázquez et al., 2016) assesses the excitatory/inhibitory nature of ICs (Martín-Vázquez et al., 2013), identifies presynaptic neuronal population (Benito et al., 2014), and analyzes its firing in relation with ICs (Fernández-Ruiz et al., 2012a,b).

Training a reservoir model with the ICs successfully replicated the various functional subtypes of task-related motor cortical neurons with relative portions similar to those obtained experimentally, except that the model exhibited a larger portion of non-task-related neurons (Figure 6). The contributions of different ICs to the generation of Hold-related and Movement-related neurons were qualitatively similar, suggesting that they play overlapping rather than specific roles in motor learning. In the modeling, we low-pass-filtered the ICs as the model is insensitive to high frequency signals. This, however, should not be interpreted as the unimportance of high-frequency oscillations to motor learning. Gamma oscillations have been implicated in cross-area communication (Colgin et al., 2009; Canolty and Knight, 2010; Yamamoto et al., 2014), and a computational study suggests that dendritic low-pass filtering makes gamma-band synchronized activity an optimal carrier of analog information between cortical neurons (Fukai, 2000).

In sum, our results indicate that there are independent components with differential layer dependence in the LFP of the primary motor cortex. These independent components show differential dynamics during self-paced voluntary movements. Interestingly, we detected a slow gamma increase after a forelimb movement in the deeper layer of the primary motor cortex, indicating a possible feedback of volitional nature of the movement. We also showed that these independent activities can be used to train a reservoir computing model to successfully replicate the motor behaviors observed experimentally, indicating active roles of these activities in motor learning.

## Author contributions

TF designed research and YI performed experiments. GM-V and TA performed experimental data analysis and TA performed modeling and simulations. GM-V, TA, and TF wrote the paper.

### Conflict of interest statement

The authors declare that the research was conducted in the absence of any commercial or financial relationships that could be construed as a potential conflict of interest.
